# Psychometric validation of the Chinese generative artificial intelligence acceptance scale among university students

**DOI:** 10.3389/fpsyg.2026.1966312

**Published:** 2026-09-29

**Authors:** Xusheng Tian, Qi Zhang

**Affiliations:** Faculty of Sport Education, Yunnan Minzu University, Kunming, China

**Keywords:** Chinese university students, cultural adaptation, generative AI acceptance, psychometric properties, scale translation

## Abstract

**Introduction:**

Generative AI use has become widespread among Chinese university students, but use does not necessarily indicate acceptance. This cross-sectional psychometric validation study adapted and evaluated the 20-item generative artificial intelligence acceptance scale for use in China.

**Methods:**

Following translation, back-translation, expert review, and pilot testing, data were collected from a convenience sample of 604 university students at six institutions in Yunnan, Anhui, and Zhejiang provinces. Participants were randomly divided into exploratory and confirmatory subsamples of 302 each.

**Results:**

Corrected item–subscale correlations ranged from 0.555 to 0.774. Exploratory factor analysis and parallel analysis supported four dimensions—performance expectancy, effort expectancy, facilitating conditions, and social influence—and the unrotated four-factor solution explained 52.22% of the total variance. The correlated four-factor CFA model fitted the second subsample well, scaled χ^2^(164) = 180.41, *p* = 0.180, robust CFI = 0.993, robust TLI = 0.992, robust RMSEA = 0.020, and SRMR = 0.035, and fitted significantly better than the three-factor alternative, Δχ^2^(3) = 102.11, *p* < 0.001. A second-order model showed comparable fit. Cronbach’s alpha ranged from 0.757 to 0.873, McDonald’s omega from 0.810 to 0.905, and composite reliability from 0.773 to 0.888. AVE ranged from 0.494 to 0.615, with performance expectancy marginally below 0.50. HTMT estimates ranged from 0.542 to 0.775, and none of their bootstrap confidence intervals included 1.00; however, the Fornell–Larcker criterion was not fully satisfied, and the upper confidence limit for PE–FC was 0.868. Measurement invariance across gender was supported.

**Conclusion:**

The findings provide preliminary support for primarily using the four subscales in similar Chinese university samples; a total score should be limited to broad descriptive use.

## 1 Introduction

Generative AI tools have become part of university study, but frequent use should not be treated as evidence of stable acceptance. In a 2025 survey of 1,041 full-time undergraduates in the United Kingdom, 92% reported using AI tools (Freeman, 2025). This contrast shows why use and acceptance need to be examined separately. In this study, generative AI acceptance refers to students’ evaluations of generative AI applications as reflected in performance expectancy, effort expectancy, facilitating conditions, and social influence. Because the original items refer to generative AI use in daily life rather than exclusively to formal academic activities, the construct is interpreted as general generative AI acceptance among university students, not acceptance confined to classroom use. Students may use an available tool for particular tasks while remaining uncertain about its accuracy, usefulness, or suitability for academic work. Research on technology acceptance also indicates that access alone does not explain adoption; beliefs about a technology’s usefulness and ease of use influence whether people intend to continue using it (Scherer et al., 2019). A measure of generative AI acceptance must therefore distinguish exposure and actual use from the judgments that support continued adoption. Without this distinction, high usage may be mistaken for trust, and differences between student groups may reflect unequal access rather than differences in acceptance.

The Unified Theory of Acceptance and Use of Technology (UTAUT) extends the central Technology Acceptance Model (TAM) emphasis on perceived usefulness and ease of use by incorporating contextual influences, including facilitating conditions and social influence (Venkatesh et al., 2003). In the present scale, these ideas are represented by performance expectancy, effort expectancy, facilitating conditions, and social influence, providing a useful basis for examining why students adopt generative AI rather than simply whether they have used it. UTAUT-based studies of university students have reported different patterns across settings. Grassini et al. (2024) applied UTAUT2 to Norwegian students, while Sergeeva et al. (2025) identified habit and performance expectancy as important predictors of adoption. In a Chinese sample, Cheng and Lu (2025) found that social influence did not significantly predict adoption. Consistent with TAM, a cross-sectional study of 685 university educators across Arab countries found that technology readiness and social influence positively predicted the perceived usefulness and effectiveness of generative AI, whereas anxiety negatively predicted both outcomes (Sallam et al., 2025). These studies mainly examined relationships between UTAUT constructs and behavioral intention. They paid less attention to whether the items used to measure those constructs retained the same meaning and structure in different student populations. This measurement question comes before prediction. If a scale does not separate the intended constructs reliably, an apparent difference between studies may result from the instrument rather than from a real difference in student acceptance. Validation is therefore necessary before scores or predictive relationships are compared across cultural and educational settings.

The instruments used in generative AI acceptance research are not yet consistent across studies. Many studies have adapted general technology acceptance scales or developed new items for specific research purposes. As a result, studies may use the same construct label while measuring it with different questions or definitions. This variation makes it difficult to determine whether differences in reported acceptance reflect the populations being studied or the instruments being used.

Karaoglan Yilmaz et al. (2024) developed the Generative Artificial Intelligence Acceptance Scale specifically for generative AI and organized it around the four core constructs of UTAUT. The scale includes 20 items across four dimensions: performance expectancy (7 items), effort expectancy (5 items), facilitating conditions (3 items), and social influence (5 items). Its fixed item set allows the same dimensions to be examined without constructing a new questionnaire for each study. Validation with 627 Turkish university students supported the reliability and four-factor structure of the original version. This provided a clearer measurement framework, but it did not establish that a translated version would function in the same way among Chinese university students.

Translating the wording of a scale does not by itself establish that the translated items measure the same constructs. A linguistically accurate item may still be interpreted differently when respondents use different technologies or study within a different educational system. Cross-cultural adaptation must therefore consider both the meaning of individual items and the structure of the scale after translation. The technological setting of Chinese higher education differs from the setting in which the original scale was validated. Chinese students may have limited access to some global AI tools such as ChatGPT and also use domestic systems, including DeepSeek, Wenxin Yiyan, and Tongyi Qianwen (Xie et al., 2025). These systems differ in their interfaces, available functions, access arrangements, and integration into academic tasks. Such differences may affect how students understand items concerning usefulness, ease of use, available support, and social expectations. Cheng and Lu (2025) applied an extended UTAUT model to explain adoption intention for DeepSeek among Chinese university students. They examined relationships among UTAUT variables rather than testing the translated Generative Artificial Intelligence Acceptance Scale. Their finding that social influence did not significantly predict adoption also shows that relationships among acceptance constructs may vary by sample or technological setting. It does not, however, establish whether the underlying measurement instrument is structurally valid.

Although the scale measures general acceptance rather than acceptance limited to formal academic activities, Chinese universities may differ in the access, guidance, training, and rules they provide for generative AI. These conditions are especially relevant to the facilitating-conditions dimension and may influence students’ responses even when the item wording is clear. The original Turkish validation cannot determine whether the same 20 items retain their four-factor structure in China. A Chinese adaptation therefore requires direct examination of item performance, factor structure, reliability, and the separation of the four constructs.

This study translated and culturally adapted the Generative Artificial Intelligence Acceptance Scale for Chinese university students. We examined item discrimination within each subscale and used empirical factor-retention criteria and exploratory factor analysis to evaluate whether the original four-factor structure could be recovered. We then tested the four-factor model against an alternative three-factor model in an independent confirmatory subsample and evaluated a second-order model to assess whether interpretation of a broad total score was defensible. Finally, we examined internal consistency, composite reliability, convergent validity, discriminant validity, and measurement invariance by gender.

The study did not propose a new acceptance model or test the causes of students’ adoption behavior. Instead, it evaluated whether the existing scale could support measurement of generative AI acceptance among Chinese university students and identified the limits that should guide its later use.

## 2 Methods

### 2.1 Participants and procedure

The Ethics Committee of Yunnan Minzu University approved the study protocol (Approval No. XYLL2026009). Before viewing the survey items, participants read an electronic information page describing the study purpose, the voluntary nature of participation, anonymity, confidentiality, and the right to leave the survey without submitting their responses. No names, student identification numbers, or contact details were collected. Participants provided electronic informed consent by selecting the agreement option. Only participants who selected this option could proceed to the questionnaire.

We used convenience sampling to recruit university students from six institutions in three provinces of China: Yunnan Minzu University and Yuxi Normal University in Yunnan Province; Zhejiang Chinese Medical University and Jinhua University of Vocational Technology in Zhejiang Province; and Huainan Normal University and Hefei University in Anhui Province. The authors sent the questionnaire link to faculty contacts at these institutions, who then distributed it to their students. Wenjuanxing was used to administer the questionnaire and record the responses. Eligible participants were currently enrolled university students aged 18 years or older who had previously used at least one generative AI tool. Participants’ academic disciplines and the duration or frequency of their prior generative AI use were not collected.

A total of 632 questionnaires were submitted. Before analysis, responses were screened for eligibility, duplicate submissions, and response quality. Duplicate submissions were identified based on repeated IP addresses, which were deleted after data screening. We excluded responses from participants with no previous generative AI experience, responses showing straightlining across the 20 scale items, and responses with an average response time of <2 s per item, a threshold informed by prior research on insufficient effort responding (Huang et al., 2012). This screening removed 28 questionnaires and left 604 for analysis. The retained questionnaires represented 95.57% of all submissions.

A computer-generated random allocation was then used to divide the 604 participants into two subsamples of equal size. Subsample 1 (*n* = 302) was used for exploratory factor analysis (EFA), and Subsample 2 (*n* = 302) was used for confirmatory factor analysis (CFA). Keeping the two analyses separate allowed the factor structure identified in one subsample to be evaluated with different observations in the other subsample. The adequacy of the achieved sample size was evaluated using both the absolute sample size and the participant-to-item ratio. Each analytic subsample contained 302 participants for 20 items, equivalent to 15.1 participants per item. This exceeded the commonly cited minimum of 200 observations for CFA and SEM and corresponded to a good absolute sample size for factor analysis according to the benchmarks summarized by Kyriazos (2018).

### 2.2 Instrument

The original English scale (Karaoglan Yilmaz et al., 2024) contains 20 items measuring four dimensions: performance expectancy (PE, 7 items), effort expectancy (EE, 5 items), facilitating conditions (FC, 3 items), and social influence (SI, 5 items). All items are rated on a 5-point Likert scale (1 = strongly disagree, 5 = strongly agree). Because several items explicitly refer to generative AI use in daily life, the scale assesses general acceptance of generative AI among university students rather than acceptance limited to academic activities.

The scale was translated and adapted in three stages. An expert in educational technology and scale development first translated the 20 items from English into Chinese. A bilingual expert then back-translated the Chinese version into English. An academic in the field of education reviewed the Chinese wording for clarity, cultural appropriateness, and consistency of terminology. Differences identified during the comparison and review were discussed until agreement was reached, and no unresolved discrepancies remained. A formal item-level or scale-level content validity index (I-CVI or S-CVI) was not calculated because the study adapted an existing validated scale without adding, deleting, or redefining its items. Instead, expert review and pilot testing were used to evaluate linguistic and conceptual equivalence, cultural appropriateness, and comprehensibility.

The draft Chinese scale was pretested with 20 university students. For each item, the students selected one of three response options: “unclear,” “generally understandable but requiring revision,” or “understood.” Feedback indicating that an item was unclear or required revision was reviewed by the research team. The resulting changes were limited to the replacement of individual words with clearer Chinese equivalents. These changes did not alter the intended meaning or construct represented by any item. No item was removed. The final 20 items and their corresponding dimensions are presented in Supplementary Table S1.

### 2.3 Statistical analysis

All analyses were performed in R version 4.4.2 using the psych (version 2.6.1), lavaan (version 0.6–21), and semTools (version 0.5–8) packages. All scale items were mandatory, and the analytic dataset contained no missing item-level responses; therefore, no missing-data estimation or imputation was required. Item means and standard deviations were calculated in the full sample. Item discrimination was evaluated within each item’s corresponding subscale. Corrected item–subscale correlations were calculated, and participants were classified into the upper and lower 27% groups based on the corresponding subscale score. Item scores were compared between the two groups using Welch’s independent-samples *t* tests.

Potential common method variance was examined using Harman’s single-factor analysis with principal axis factoring. The variance explained by the first unrotated factor was compared with the commonly used 40% reference value. A one-factor confirmatory model was also fitted to assess whether all 20 items could be represented adequately by a single latent factor. These procedures were treated as diagnostic checks rather than proof that common method variance was absent.

EFA was conducted in Subsample 1 using principal axis factoring based on the Pearson correlation matrix. Sampling adequacy and factorability were examined using the Kaiser–Meyer–Olkin measure and Bartlett’s test of sphericity. Factor retention was evaluated using parallel analyses based on both Pearson and polychoric correlation matrices, together with Velicer’s minimum average partial (MAP) tests. Because the study evaluated the structure proposed for the original scale, a four-factor solution was examined using Promax rotation. The decision to retain four factors considered both the original theoretical structure and the empirical factor-retention evidence. Loadings with an absolute value of 0.30 or higher were reported, including meaningful cross-loadings. Total variance explained was calculated from the unrotated four-factor solution rather than by summing the variances of the correlated factors after Promax rotation.

CFA was conducted in Subsample 2. The primary model specified four correlated first-order factors: PE, EE, FC, and SI. An alternative three-factor model combined PE and EE while retaining FC and SI as separate factors. A second-order model specified PE, EE, FC, and SI as first-order factors loading on a higher-order general acceptance factor. The three models were estimated using robust maximum likelihood (MLR). Model fit was evaluated using the scaled χ^2^ statistic with its degrees of freedom and *p* value, robust CFI, robust TLI, robust RMSEA with its 90% confidence interval, and SRMR. Nested models were compared using robust scaled χ^2^ difference tests. Standardized factor loadings, standard errors, z values, and *p* values were reported. No residual covariances were added after estimation, and modification indices were not used to respecify the models.

Internal consistency was evaluated separately for each subscale using Cronbach’s alpha, McDonald’s omega, and composite reliability. Values of 0.70 or higher were treated as acceptable. Convergent validity was examined using average variance extracted (AVE), with 0.50 used as the reference value. Discriminant validity was assessed using the Fornell–Larcker criterion (Fornell and Larcker, 1981) and the heterotrait–monotrait ratio of correlations (HTMT). Ninety-five percent bootstrap confidence intervals for the HTMT estimates were calculated using 2,000 resamples, and HTMT values below 0.85 were used as the reference criterion.

Measurement invariance of the four-factor model across gender was examined in the full sample using multigroup CFA. Configural, metric, scalar, and strict invariance models were fitted sequentially. Invariance was evaluated primarily using changes in approximate fit indices, with ΔCFI ≤0.010 and ΔRMSEA ≤0.015 indicating invariance. Changes in SRMR were also examined, using thresholds of ≤0.030 for metric invariance and ≤0.010 for scalar and strict invariance (Chen, 2007).

## 3 Results

The sociodemographic characteristics of the participants and the generative AI tools they had used are summarized in Table 1.

**Table 1:** Sociodemographic characteristics of the participants (*n* = 604).

Characteristic	Category	*n*	%
Gender	Male	416	68.87
	Female	188	31.13
Age	18–22 years	443	73.34
	23–25 years	91	15.07
	26–30 years	39	6.46
	31–35 years	25	4.14
	≥36 years	6	0.99
Academic level	Undergraduate	407	67.38
	Master’s student	160	26.49
	Doctoral student	37	6.13
Generative AI tools usedᵃ	Doubao	428	70.86
	ChatGPT	307	50.83
	DeepSeek	305	50.50
	Tongyi Qianwen	261	43.21
	iFlytek Spark	108	17.88
	Kimi	102	16.89
	Gemini	94	15.56
	Claude	93	15.40
	ChatGLM	91	15.07
	Wenxin Yiyan	90	14.90
	Other generative AI tools	85	14.07
	Tencent Hunyuan	80	13.25

Age was recorded categorically. ᵃParticipants could select more than one generative AI tool they had used; therefore, percentages for generative AI tools do not sum to 100%.

### 3.1 Item analysis

Item discrimination was examined using corrected item–subscale correlations and comparisons between the upper and lower 27% groups formed from the corresponding subscale scores. Corrected item–subscale correlations ranged from 0.555 to 0.774. All items differed significantly between the upper and lower groups (all *p* < 0.001), with t values ranging from 19.15 to 26.06. These results indicated adequate discrimination for all 20 items, which were therefore retained for factor analysis. Detailed item-level results are presented in Supplementary Table S2.

### 3.2 Common method variance

Two diagnostic analyses were used to examine whether the responses were dominated by a single factor. The first unrotated factor accounted for 38.67% of the variance. A one-factor CFA also fitted the data poorly (CFI = 0.768, RMSEA = 0.119). Taken together, these results did not indicate that one general factor accounted for the observed four-dimensional pattern.

### 3.3 Exploratory factor analysis

The data from Subsample 1 (*n* = 302) were suitable for factor analysis. The KMO value was 0.928, and Bartlett’s test of sphericity was significant, χ^2^(190) = 2637.52, *p* < 0.001. Parallel analyses based on both Pearson and polychoric correlation matrices supported a four-factor solution. The MAP test based on the polychoric correlation matrix also supported four factors, whereas the Pearson-based MAP test suggested three factors. Considering the theoretical structure of the original scale, the parallel analysis results, and the subsequent CFA model comparison, the four-factor solution was retained.

The four-factor solution explained 52.22% of the total variance. As shown in Table 2, primary factor loadings ranged from 0.559 to 0.803 for PE, 0.662 to 0.784 for EE, 0.563 to 0.796 for FC, and 0.579 to 0.839 for SI. All nonprimary loadings had absolute values below 0.30, indicating that no item showed a salient cross-loading. All 20 items were therefore retained.

**Table 2:** Exploratory factor analysis loadings (*n* = 302).

Item	PE	EE	FC	SI
I1	0.565			
I2	0.559			
I3	0.681			
I4	0.803			
I5	0.641			
I6	0.643			
I7	0.749			
I8		0.760		
I9		0.784		
I10		0.721		
I11		0.667		
I12		0.662		
I13			0.796	
I14			0.563	
I15			0.725	
I16				0.685
I17				0.727
I18				0.780
I19				0.579
I20				0.839

Principal-axis factoring with Promax rotation was used. Loadings with absolute values below 0.30 are suppressed. PE, performance expectancy; EE, effort expectancy; FC, facilitating conditions; SI, social influence.

### 3.4 Confirmatory factor analysis

CFA was conducted in Subsample 2 (*n* = 302). The correlated four-factor model showed good fit to the data, scaled χ^2^(164) = 180.41, *p* = 0.180, robust CFI = 0.993, robust TLI = 0.992, robust RMSEA = 0.020, 90% CI [0.000, 0.035], and SRMR = 0.035. In comparison, the three-factor model combining PE and EE showed poorer fit, scaled χ^2^(167) = 339.84, *p* < 0.001, robust CFI = 0.929, robust TLI = 0.919, robust RMSEA = 0.064, 90% CI [0.054, 0.073], and SRMR = 0.054. The scaled chi-square difference test indicated that the three-factor model fitted significantly worse than the correlated four-factor model, Δχ^2^(3) = 102.11, *p* < 0.001.

The second-order model also showed good fit, scaled χ^2^(166) = 183.06, *p* = 0.173, robust CFI = 0.993, robust TLI = 0.992, robust RMSEA = 0.020, 90% CI [0.000, 0.035], and SRMR = 0.036. Its fit did not differ significantly from that of the correlated four-factor model, Δχ^2^(2) = 2.86, *p* = 0.239. Because the primary purpose of the study was to evaluate the four original dimensions as distinguishable constructs, the correlated first-order four-factor model was retained as the principal measurement model. Model-fit results are presented in Table 3.

**Table 3:** Confirmatory factor analysis fit indices (*n* = 302).

Model	Scaled χ^2^	df	*p*	Robust CFI	Robust TLI	Robust RMSEA	90% CI	SRMR
Three-factor	339.84	167	< 0.001	0.929	0.919	0.064	[0.054, 0.073]	0.054
Four-factor	180.41	164	0.180	0.993	0.992	0.020	[0.000, 0.035]	0.035
Second-order	183.06	166	0.173	0.993	0.992	0.020	[0.000, 0.035]	0.036

Models were estimated using robust maximum likelihood (MLR). CFI, comparative fit index; TLI, Tucker–Lewis index; RMSEA, root mean square error of approximation; SRMR, standardized root mean square residual.

Standardized factor loadings for the retained correlated four-factor model ranged from 0.629 to 0.852 and were all statistically significant (all *p* < 0.001; Table 4). The standardized solution is presented in Figure 1.

**Table 4:** Standardized CFA factor loadings.

Factor	Item	Standardized loading	SE	z	*p*	95% CI
PE	I1	0.629	0.048	13.08	<0.001	[0.535, 0.723]
PE	I2	0.658	0.039	16.85	<0.001	[0.581, 0.734]
PE	I3	0.674	0.040	17.06	<0.001	[0.597, 0.752]
PE	I4	0.738	0.033	22.54	<0.001	[0.674, 0.802]
PE	I5	0.699	0.032	21.58	<0.001	[0.636, 0.763]
PE	I6	0.720	0.037	19.23	<0.001	[0.646, 0.793]
PE	I7	0.791	0.033	23.75	<0.001	[0.726, 0.856]
EE	I8	0.757	0.042	18.07	<0.001	[0.675, 0.839]
EE	I9	0.703	0.041	17.14	<0.001	[0.623, 0.783]
EE	I10	0.773	0.036	21.31	<0.001	[0.702, 0.844]
EE	I11	0.631	0.048	13.19	<0.001	[0.537, 0.725]
EE	I12	0.740	0.035	21.21	<0.001	[0.672, 0.809]
FC	I13	0.751	0.042	17.74	<0.001	[0.668, 0.834]
FC	I14	0.741	0.036	20.83	<0.001	[0.671, 0.810]
FC	I15	0.695	0.043	16.01	<0.001	[0.610, 0.780]
SI	I16	0.810	0.032	25.17	<0.001	[0.747, 0.873]
SI	I17	0.743	0.036	20.63	<0.001	[0.672, 0.813]
SI	I18	0.758	0.036	20.78	<0.001	[0.687, 0.830]
SI	I19	0.752	0.035	21.28	<0.001	[0.683, 0.822]
SI	I20	0.852	0.028	30.50	<0.001	[0.797, 0.907]

PE, performance expectancy; EE, effort expectancy; FC, facilitating conditions; SI, social influence; SE, standard error; CI, confidence interval.

**Figure 1:**
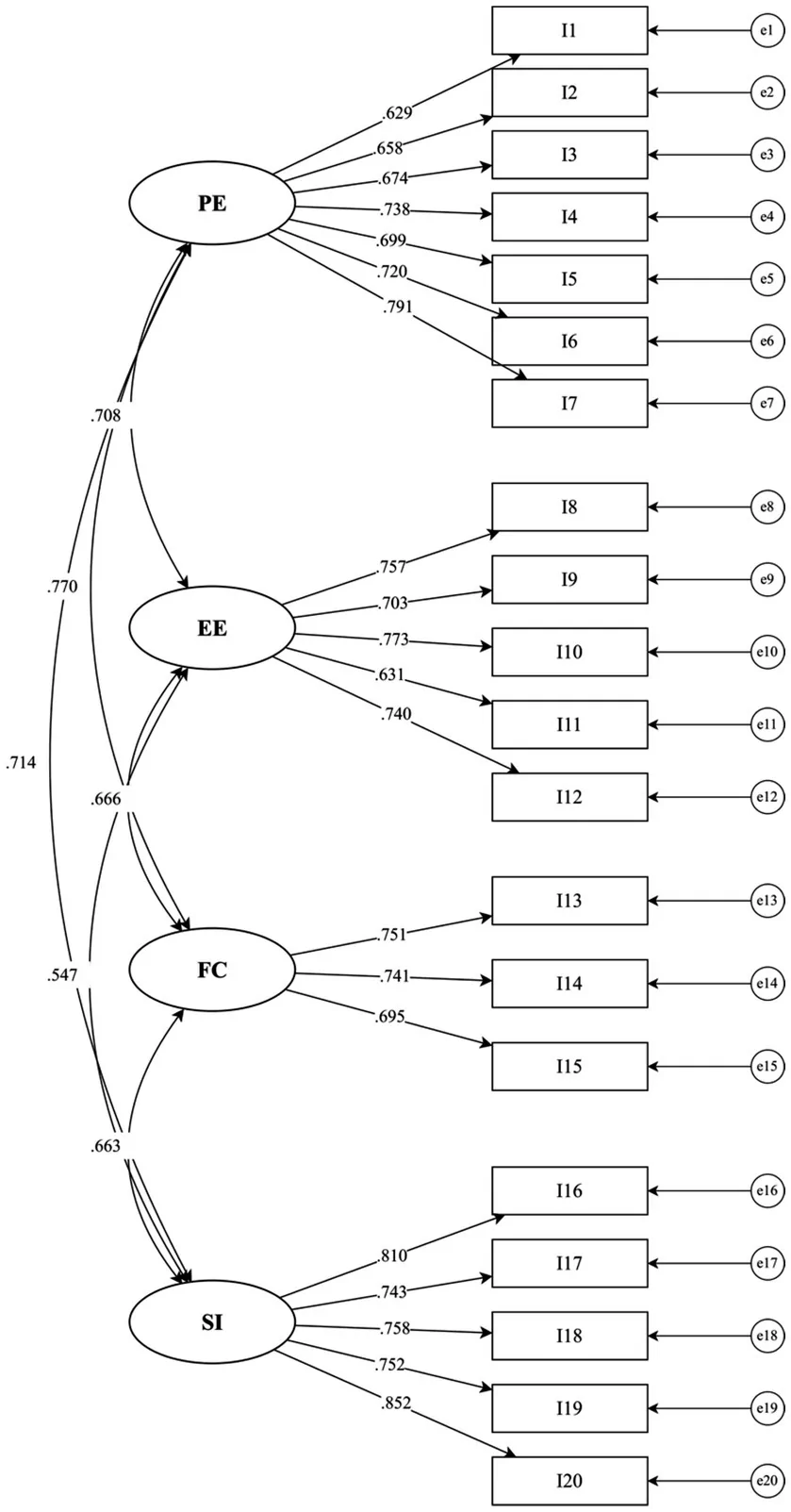
Standardized solution for the correlated four-factor confirmatory factor analysis model.

### 3.5 Reliability and convergent validity

Internal consistency was acceptable for all four dimensions (Table 5). Cronbach’s alpha coefficients ranged from 0.757 to 0.873, ordinal McDonald’s omega coefficients ranged from 0.810 to 0.905, and composite reliability values ranged from 0.773 to 0.888. All these estimates exceeded the 0.70 reference value.

**Table 5:** Reliability and convergent validity.

Dimension	Items	Cronbach’s α	Ordinal McDonald’s ω	CR	AVE	√AVE
PE	7	0.867	0.901	0.872	0.494	0.703
EE	5	0.847	0.880	0.845	0.522	0.722
FC	3	0.757	0.810	0.773	0.532	0.729
SI	5	0.873	0.905	0.888	0.615	0.784

Cronbach’s α and ordinal McDonald’s ω were calculated using the full sample (*N* = 604), whereas CR and AVE were derived from the CFA subsample (*n* = 302). CR = composite reliability; AVE = average variance extracted; √AVE = square root of AVE.

The AVE values were 0.494 for PE, 0.522 for EE, 0.532 for FC, and 0.615 for SI. The values for EE, FC, and SI exceeded the 0.50 reference value. The AVE for PE was marginally below 0.50, although its composite reliability (0.872), Cronbach’s alpha (0.867), and McDonald’s omega (0.901) were acceptable. Thus, convergent validity was supported for EE, FC, and SI, whereas the evidence for PE was marginal but remained acceptable when considered together with its reliability estimates.

### 3.6 Discriminant validity

Discriminant validity was evaluated using the Fornell–Larcker criterion and the heterotrait–monotrait ratio of correlations (HTMT). As shown in Table 6, the Fornell–Larcker criterion was not fully satisfied. The square root of AVE for PE (0.703) was lower than its correlations with EE (0.708), FC (0.770), and SI (0.714). For comparisons that did not involve PE, the interfactor correlations were lower than the corresponding square roots of AVE.

**Table 6:** Discriminant validity: Fornell–Larcker criterion and HTMT.

Construct	PE	EE	FC	SI
PE	**0.703**	0.703	0.775	0.713
EE	0.708	**0.722**	0.665	0.542
FC	0.770	0.666	**0.729**	0.669
SI	0.714	0.547	0.663	**0.784**

Diagonal values in bold are the square roots of AVE; values below the diagonal are latent factor correlations; values above the diagonal are Pearson HTMT estimates. PE = performance expectancy; EE = effort expectancy; FC = facilitating conditions; SI = social influence.

Pearson HTMT values ranged from 0.542 to 0.775, and all point estimates were below the 0.85 reference value. None of the 95% bootstrap confidence intervals included 1.00. However, the upper confidence limit for the PE–FC comparison was 0.868, slightly exceeding the 0.85 reference value. Taken together, the results provided qualified rather than unequivocal support for discriminant validity, with the greatest overlap observed between PE and FC. Complete HTMT estimates and bootstrap confidence intervals are presented in Supplementary Table S3.

### 3.7 Measurement invariance across gender

Multigroup CFA was conducted to examine measurement invariance across gender (Supplementary Table S4). The configural model showed good fit (robust CFI = 0.985, TLI = 0.983, RMSEA = 0.029, and SRMR = 0.033). Changes in CFI, RMSEA, and SRMR remained within the prespecified cutoffs at the metric, scalar, and strict levels. These results supported strict measurement invariance across gender.

## 4 Discussion

The Chinese adaptation of the scale developed by Karaoglan Yilmaz et al. (2024) retained all 20 items and supported a four-dimensional structure comprising performance expectancy, effort expectancy, facilitating conditions, and social influence. The factor pattern identified in the EFA was supported by the CFA conducted in an independent subsample, and all four subscales showed acceptable internal consistency. Convergent validity was supported for EE, FC, and SI, whereas the AVE for PE was marginally below the conventional reference value. Evidence for discriminant validity was qualified rather than unequivocal, particularly for PE and FC. Preliminary measurement invariance results nevertheless indicated that the four-factor structure operated similarly across male and female participants. Overall, the findings support the use of the four related subscales in university student samples similar to the one examined here.

### 4.1 Psychometric properties

The EFA supported the four-dimensional arrangement reported for the original instrument. Each item had its strongest loading on its theoretically assigned dimension, and all nonprimary loadings were below 0.30. The translated items did not show salient shifts in factor membership or problematic cross-loadings. Parallel analyses based on Pearson and polychoric correlations also supported four factors, although the Pearson-based MAP test suggested three factors. The four-factor interpretation should therefore be viewed as supported by the overall pattern of evidence rather than uniformly selected by every factor-retention method.

The CFA offered a stricter test because it was conducted with observations that had not been used in the EFA. The four-factor model fitted these data well and performed better than the alternative model in which PE and EE were combined. No post-hoc residual covariances were added, so the reported fit did not depend on sample-specific model adjustments. The two analyses therefore provide complementary evidence. The EFA shows that the expected item pattern can be recovered, while the CFA reproduces that pattern in a separate subsample. This procedure provides internal replication within the overall sample, but it should not be treated as external validation in a new population.

The second-order model also fitted the data well and did not differ significantly from the correlated first-order four-factor model. This result provides limited empirical support for describing a broader generative AI acceptance construct. Nevertheless, the correlated first-order model was retained as the principal measurement model because it preserves the four theoretically defined dimensions of the original instrument (Karaoglan Yilmaz et al., 2024). Accordingly, a total score may be used as a broad descriptive indicator, but it should not replace interpretation of the four subscale scores.

The unrotated four-factor principal-axis factoring solution explained 52.22% of the total item variance, which was lower than the proportion reported in the original study. This difference does not necessarily indicate a weaker factor structure because the two studies used different extraction methods. Karaoglan Yilmaz et al. (2024) used principal component analysis, which represents total observed variance, whereas principal-axis factoring estimates latent factors primarily from the variance shared among items and commonly produces lower percentages of explained variance (Fabrigar et al., 1999). Because Promax is an oblique rotation and the rotated factors share variance, the percentage reported in the present study was obtained from the unrotated solution rather than by summing the rotated factor contributions.

The reliability estimates were consistent with the structural results. Cronbach’s alpha coefficients ranged from 0.757 to 0.873, ordinal McDonald’s omega coefficients ranged from 0.810 to 0.905, and composite reliability values ranged from 0.773 to 0.888 across the four dimensions. The AVE values for EE, FC, and SI exceeded 0.50, whereas the AVE for PE was 0.494. The PE value was only marginally below the conventional reference value and was accompanied by acceptable alpha, omega, and composite reliability. Accordingly, the evidence for PE convergent validity should be described as marginal rather than as clearly inadequate or fully established.

### 4.2 Applicability in the Chinese context

Adapting the scale for Chinese students involved more than translating its wording. The instrument was tested in a technological setting where access to some global AI services is limited and domestic systems are widely used. These systems include DeepSeek, Wenxin Yiyan, and Tongyi Qianwen (Xie et al., 2025). Within this setting, students’ responses were still organized around the four dimensions identified in the Turkish validation by Karaoglan Yilmaz et al. (2024). They distinguished the expected benefits of generative AI, the effort required to use it, the resources available to support its use, and the influence of people around them. The support for this structure suggests that the four concepts remain meaningful in the Chinese university context. Accordingly, the scale should be interpreted as assessing general acceptance of generative AI among university students rather than acceptance confined specifically to academic learning activities.

Chinese studies have mainly used UTAUT constructs to explain behavioral intention toward generative AI (Cao et al., 2025; Tang et al., 2025). Such analyses depend on the assumption that translated measures continue to represent their intended constructs. Predictive relationships are difficult to interpret if that assumption has not first been examined. The present study addressed this measurement question by evaluating the item structure, reliability, and construct validity of the Chinese version. The findings support its preliminary use for examining separate aspects of generative AI acceptance in university student samples similar to the one studied here. Preliminary measurement invariance was supported across gender; however, comparisons across academic levels, provinces, institutions, or AI platforms would require additional invariance testing.

Consistent with the construct definitions of the original scale (Karaoglan Yilmaz et al., 2024), the four subscales have distinct practical meanings. PE reflects students’ perceptions of the usefulness and benefits of generative AI; EE reflects the perceived ease of learning and using these tools; FC reflects access to compatible technologies, relevant information, and interpersonal support; and SI reflects the expectations and behavior of important others. The subscale scores can therefore help identify whether acceptance is primarily associated with perceived usefulness, usability, available support, or the social environment. They should be interpreted separately rather than replaced by an undifferentiated total score.

### 4.3 Limitations and future directions

The convenience sampling design limits the generalizability of the findings. Participants were recruited through faculty contacts at six institutions in Yunnan, Anhui, and Zhejiang Provinces rather than through population-based sampling. Although the sample size was adequate for EFA and CFA (Kyriazos, 2018), an adequate sample size does not eliminate selection bias. The gender distribution was also imbalanced, which remains relevant because gender may be associated with technology acceptance (Venkatesh et al., 2003). Preliminary measurement invariance was supported across gender, but invariance across academic levels, provinces, institutions, and disciplines was not examined. Participants’ academic disciplines and the duration or frequency of their generative AI use were also not collected. Future studies should therefore use more balanced and diverse samples.

The types of generative AI tools that participants had used were described, but their proficiency and intensity of use were not measured. Moreover, because several items refer to generative AI use in daily life, the scale assesses general acceptance among university students rather than acceptance limited specifically to educational use. No external measures of actual AI use, AI literacy, behavioral intention, or learning outcomes were collected. The present findings therefore provide evidence regarding internal structure and reliability, but not criterion, predictive, or nomological validity.

The study used a single administration of a self-report questionnaire. Harman’s single-factor analysis and the one-factor CFA did not suggest that one general factor dominated the responses, but these diagnostic procedures cannot eliminate all forms of common method or response bias. A single administration also cannot establish temporal stability. Future research should use external data sources, test–retest designs, and longitudinal assessments.

Some psychometric issues also require further investigation. Although most factor-retention evidence supported four factors, the primary EFA was theory-guided and the Pearson-based MAP test suggested three factors. FC contained only three items and showed comparatively lower reliability, while the AVE for PE was marginally below the conventional reference value. In addition, the Fornell–Larcker criterion was not fully satisfied, and the PE–FC HTMT confidence interval slightly exceeded the strict 0.85 reference value. These findings do not require the factors to be merged, because the four-factor CFA model fitted significantly better than the three-factor alternative, but they indicate that the subscales should remain the primary basis for interpretation. A total score should be used only as a broad descriptive indicator until the structure is replicated in independent samples.

## 5 Conclusion

Using responses from 604 university students, this study evaluated the psychometric properties of a 20-item Chinese adaptation of the Generative Artificial Intelligence Acceptance Scale. Analyses conducted in separate EFA and CFA subsamples supported four related dimensions: performance expectancy, effort expectancy, facilitating conditions, and social influence. All four subscales demonstrated acceptable internal consistency. Convergent validity was supported for effort expectancy, facilitating conditions, and social influence, whereas the evidence for performance expectancy was marginal. HTMT results generally supported the distinction among the four dimensions, although the Fornell–Larcker criterion was not fully satisfied and the PE–FC comparison warrants particular caution.

The findings support the preliminary use of the instrument primarily through its four subscale scores in Chinese university student samples similar to the one examined here. Preliminary measurement invariance was supported across gender. The acceptable second-order model permits a total score to be reported as a broad descriptive indicator, but it should not replace interpretation of the four dimensions. Further research should examine measurement invariance across academic levels, regions, and institution types and evaluate criterion-related validity, predictive validity, and test–retest reliability.

## Data Availability

The raw data supporting the conclusions of this article will be made available by the authors, without undue reservation. Requests to access the datasets should be directed to the corresponding author.
